# The history, evolution and basic science of osteotomy techniques

**DOI:** 10.1007/s11751-017-0296-4

**Published:** 2017-10-06

**Authors:** John Dabis, Oliver Templeton-Ward, Alice E. Lacey, Badri Narayan, Alex Trompeter

**Affiliations:** 1 St. Georges University Hospital, Blackshaw Road, Tooting, London, SW17 0QT UK; 2 Royal Surrey County Hospital, Egerton Road, Guildford, Surrey, GU2 7XX UK; 3 Health Education England (NW), 3 Piccadilly, Manchester, M1 3BN UK; 4 Royal Liverpool and Broadgreen University Hospital, Broadgreen Hospital, Liverpool, L13 4LB UK

**Keywords:** Osteotomy, Bone regenerate, Drill and osteotome, Gigli saw, Distraction osteogenesis, Ilizarov

## Abstract

Osteotomy techniques date back to Hippocrates circa 415 BC (Jones Hippocrates collected works I, Harvard University Press, Cambridge, 2006; Brorson in Clin Orthop Relat Res 467(7):1907–1914, 2009). There is debate about the best way to divide the bone surgically and which technique yields the best bone regenerate in lengthening; ensuring predictable new bone formation and healing of the osteotomy are the primary goals. We review the history and techniques of the osteotomy and consider the evidence for optimum bone formation. Methods discussed include variants of the ‘drill and osteotome’ technique, use of the Gigli saw and use of a power saw. Differences in bone formation through the different techniques are covered.

## Introduction

Osteotomies are performed broadly for two purposes: a simple osteotomy to acutely realign the axis of the bone and that which allows bone lengthening or bone transport. A simple osteotomy is used to correct angular or rotational deformities where healing is in compression or, in the case of opening wedge osteotomies, callus is required to fill a gap [3]. The former relies on stability to promote union in the new position, whereas, in the latter, healing is more difficult as an element of instability is introduced. In bone lengthening and transport, the technique leads to formation of new bone over a segment; the methods of bone division are critical to provide a quality regenerate [4, 5].

Which osteotomy technique yields the best bone regenerate? We review the current evidence supporting each technique; the history, basic science and different methods of osteotomy are presented with their advantages and disadvantages to ascertain if there is a preferred method for the desired outcome.

## The history of osteotomy

In the Edwin Smith papyrus, a document from Egypt circa 1600 BC, three cases of humeral fractures are described and the importance of bone alignment to prevent deformity is expressed [2]. In the Hippocratic Corpus ‘De Fracturis’ (circa 415 BC) [2] mention is made of using a new traumatic fracture to aid with improving the alignment of a previously angulated humerus. Following this observation, Hippocrates developed a device known as the Hippocratic Scamnum, a traction device used to realign bones as they healed [2]. Through the times of the Romans and Greeks, physicians such as Celsus and Galen advanced the management of fractures [6, 7]. This evolved use of reduction techniques often with the help of the Hippocratic Scamnum. However, it took until the sixteenth century for the use of deliberate closed fracturing of bones to aid correction of deformity [8]. This process was then known as osteoclasis or osteoclasia, from the Greek ‘*osteo*’ meaning bone, and ‘*klasis*’ meaning break.

More recently, Langenbeck described performing planned open osteotomies based on his experience in the Schleswig–Holstein war (1848–1850) [9, 10]. He used a straight pointed saw to debride dead bone from bullet wounds. Unfortunately, infection rates were high as there was little in the way of asepsis and large open approaches were used. Towards the latter part of the nineteenth century, surgeons began to develop instruments that allowed them to perform what became known as ‘subcutaneous osteotomy’ [10]. This yielded markedly lower complication rates.

In 1879 William Adams, in the British Medical Journal ‘on subcutaneous osteotomy’, [2] detailed his and other surgeons’ experiences with techniques in both the upper and lower limbs. Adams points out that ‘section or partial section of the lower end of the femur with a chisel’ whilst ‘using Lister’s aseptic technique’ has been ‘very successfully adopted’ by Professor MacEwen in Glasgow for the treatment of knee ankylosis. In 1880, MacEwen published the first book devoted entirely to osteotomy where he detailed his experience of 1800 cases with few complications [11]. The dissemination of these techniques using improved instruments and aseptic precautions led to the increasing popularity of osteotomy for deformity correction [12].

During the twentieth century, attempts were made to transfer the osteotomy techniques for the treatment of arthritis. Brittain, in the UK, published on use of a distal femoral osteotomy to treat valgus knees with isolated lateral compartment arthritis [13], whilst in 1941, Wardle began performing a high tibial osteotomy for the same indications. He noted ‘complete relief of pain in all his patients’ and very few significant complications. Surgeons realised that angular osteotomies with subsequent stabilisation would heal and provide correction of limb deformity or alter the load distribution across joints [14, 15].

At around the same time in Russia, Ilizarov was perfecting his use of the external fixator to improve bone healing and the treatment of fractures; in his dissertation ‘Transosseous compression osteosynthesis by the authors apparatus’, Ilizarov described his clinical results of 444 patients after arthrodesis, correction osteotomies, non-union and fracture treatment [16]. He formulated his principles for optimisation of bone healing: preservation of the blood supply and osteogenic tissue, accurate reduction, stable fixation, functional activity of the muscles and joints and early patient mobilisation [16]. The ability to regenerate bone under distraction was discovered serendipitously some years later; confirmation of this phenomenon, in subsequent experiments, introduced bone lengthening by ‘distraction osteogenesis’ into his practice [16]. Other surgeons around that time began to publish their experiences of limb lengthening also [17].

## Basic science of bone healing and formation

Knowledge of fracture healing has advanced; the complex and pivotal interplay of biology and biomechanics is recognised and the management of fractures and, consequently, osteotomies have evolved. Perren acknowledged the concept of biological fixation after realising the important role biology played in the management of fractures [18]. Two broad types of bone healing are recognised: primary (direct) bone healing without formation of callus; and secondary (indirect) bone healing which consists of both endochondral and intramembranous ossification [19]. Secondary bone healing relies on the sequential steps of tissue differentiation, resorption of the fracture edges and union of the fragments by callus before remodelling recreates the original Haversian system. Primary bone healing occurs in situations where bone ends are anatomically reduced and an environment of absolute stability minimises interfragmentary strain enabling osteons to cross the fracture at the compressed surface [18]. The manner of fracture or osteotomy stabilisation will influence the strain environment and subsequently the way bone is formed in the gap.

## Osteotomy healing in compression

Osteotomies that are closing wedges are akin to optimally reduced fractures. In a low-strain and rigidly stabilised environment, primary bone healing is achieved; the gap between bone ends is less than 0.01 mm and interfragmentary strain is less than 2%, permitting contact-healing to occur. Cutting cones from osteoclasts cross the osteotomy site and create a template for osteoblasts at the rear to lay down new bone. Union and restoration of the Haversian system occur in concert and lamellar bone is formed by direct remodelling [20]. In certain circumstances, a gap between bony surfaces of more than 0.01 mm may exist in which case gap-healing occurs and lamellar bone is laid down between the bone ends, perpendicular to the normal Haversian system. This then undergoes secondary remodelling by cutting cones to orientate itself to the mechanical stress placed on the bone [21].

## Osteotomy healing without full bony apposition

In some clinical situations closing or shortening osteotomies are undesirable; opening wedge osteotomies can be performed in order to correct angular deformities, alter the mechanical axis and without loss of length. The evolution of this technique is illustrated in the knee; osteotomy surgery for medial unicompartmental osteoarthritis of the knee focused on a laterally based, closing wedge high tibial osteotomy as popularised by Coventry [22–25]. There were some disadvantages including under correction, peroneal nerve injury, damage to the proximal tibiofibular joint and the loss of bone stock [26, 27]. The use of a medially based opening wedge osteotomy was theoretically simpler but presented problems with bone healing [28]. The creation of a large void imparted instability to the osteotomy and initial implants were unable to withstand the axial and rotational forces across the proximal tibia for long enough to allow bone healing [27, 29]. Use of fixed angle devices (such as the Tomofix™ plate, Synthes, Switzerland) appears to have optimised the balance between stability and micro-motion. This creates a strain environment favourable for bone formation at the osteotomy site [30]. Staubli has studied the mode of bone healing in opening wedge osteotomy and found that gap filling occurs from apex to base on sequential X rays, presumably influenced by the differing strain environments [31]. One theory is the strain environment is too low at the base, directly under the plate, to stimulate any bone healing, as the fracture gap is too large. At the apex of the opening wedge, the fracture gap is small and hence has a higher strain environment. The precise mode of bone formation in this situation is unclear, but it is likely that endochondral ossification plays a major role. It has been shown on CT that full progression to mineralisation is not evident in the majority of cases until 1 year post-operatively [25]. Gaps of up to 20 mm can be filled successfully with new bone formation without use of any grafting material [32].

## Osteotomy healing in distraction: bone transport

‘Distraction osteogenesis is a biological process of new bone formation between the surfaces of bone segments that are gradually separated by incremental traction’ [33]. Traditionally, it is a continuum divided into five distinct periods: osteotomy, latency, distraction, consolidation and remodelling. The first step in any limb-lengthening procedure is to perform an osteotomy at the desired location to allow distraction. The osteotomy initiates the haematoma formation, which becomes a scaffold for callus formation [34]. Within the latency phase, the inflammatory response initiates chemotactic and angiogenic factors that promote new vessel formation and differentiation of osteoblasts to osteoclasts. The formation of cartilaginous callus is one of the features of this healing process and occurs by both endochondral and intramembranous pathways [20].

The haematoma allows for fibrin-rich granulation tissue to form and to transform from soft to hard callus. The early phases of this process are those of fracture healing whilst the succeeding process of bone lengthening and consolidation differ histologically [35, 36]. Avascular fibrous tissue (the Fibrous Inter Zone—FIZ) fills the gap between the cut surfaces of the bone; the areas either side of the FIZ have vascular sinusoids. Osteoid tendrils, created by a group of osteoblasts, protrude into this zone if distraction is applied and form a primary mineralisation fronts (PMF). Over time the tendrils elongate in the process of micro-column formation (MCF), eventually bridging across the entire FIZ. When distraction is discontinued at the site of osteotomy, the micro-columns remodel and form lamellar bone [35, 36].

The mode of new bone formation in distraction osteogenesis is debated. Ilizarov has stated bone is formed by intramembranous ossification but small islands of endochondral ossification are witnessed when mechanical instability of the distraction device is suboptimal. There are animal studies (Forriol [37] and Peltonen et al. [38]) demonstrating a combination of intramembranous and endochondral ossification occurring simultaneously at the site of distraction.

## Techniques for osteotomy for distraction osteogenesis

Several osteotomy methods are used and, in the setting of limb reconstruction and distraction osteogenesis, the technique can have a significant impact on the successful creation of new bone. The preconditions for successful distraction osteogenesis include minimal trauma at the osteotomy, good blood supply, satisfactory stability of the fixation method and rhythmical distraction at an appropriate rate [36]. Conditions that degrade local or regional blood supply have a detrimental effect on new bone formation. Hence, the techniques have evolved to minimise local trauma, thermal necrosis and disruption to the blood supply at the osteotomy site.

## Evolution of bone division techniques

### Corticotomy

**Fig. 1:**
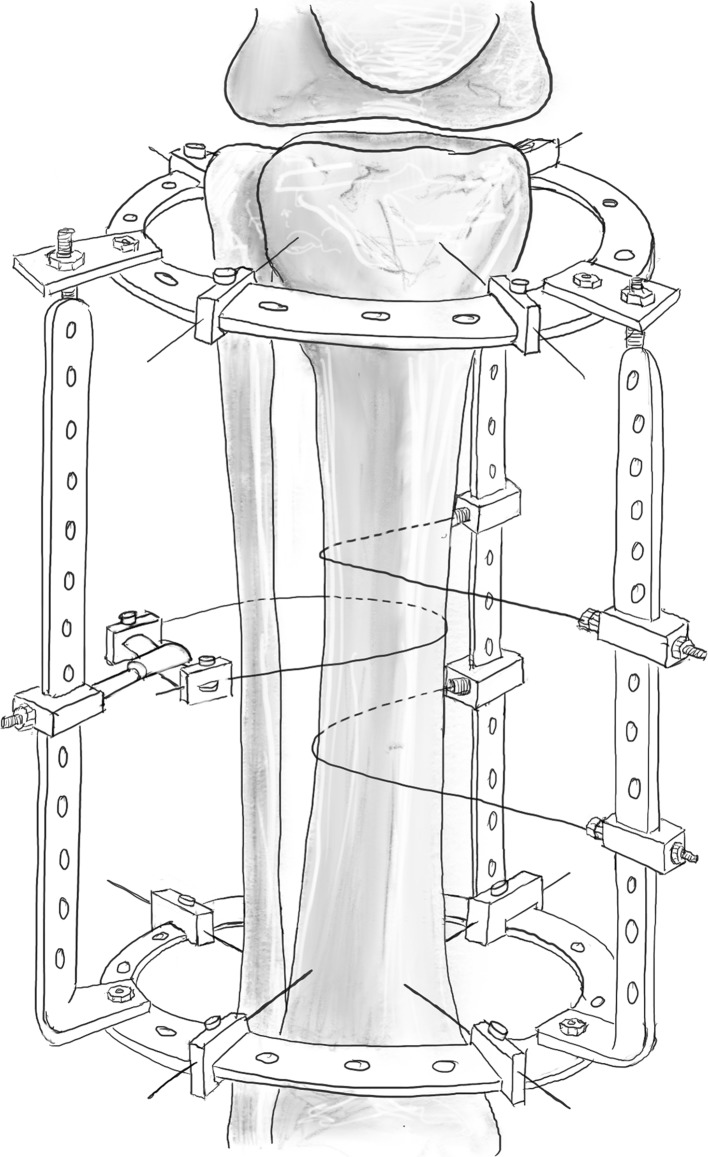
Illustration showing Ilizarov’s original non-invasive method of corticotomy using tensioned wires. Redrawn from original illustration in Tranosseous Oseosynthesis, Ilizarov GA (1992), Springer

Ilizarov first described corticotomy as a means of surgical division of the bone as a low-energy osteotomy of the cortex (Fig. 1). This technique was based on interrupting the cortex of the long bone whilst not violating the medullary vascularity or the periosteum. He believed that preservation of the medullary circulatory system enhanced bone formation and was necessary for successful osteogenesis [39]. An osteotome was used to complete this division, but was associated with an increased risk of propagating the simple osteotomy to a complex, multi-fragmentary fracture with displacement. It is also difficult to perform in small bones [40].

**Fig. 2:**
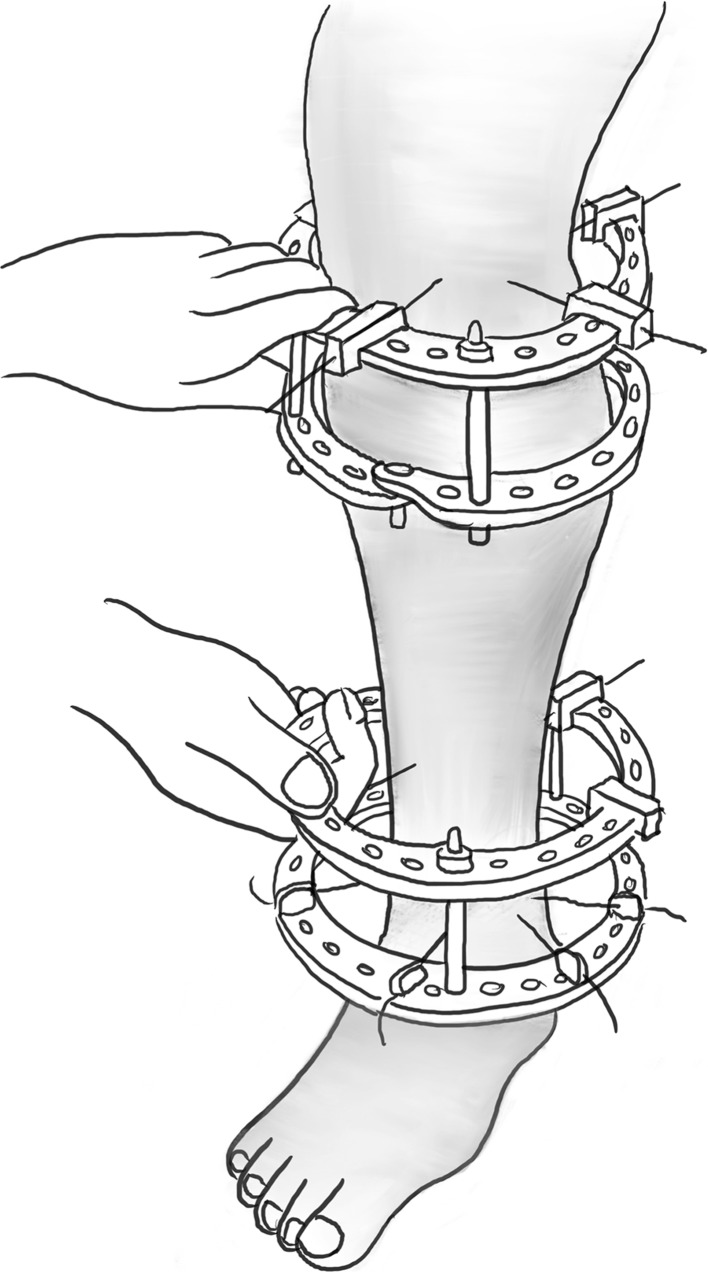
An example of rotational osteoclasis, which requires the rings to be attached, yet no interconnecting rods. Redrawn from original illustration in Tranosseous Oseosynthesis, Ilizarov GA (1992), Springer

In the Ilizarov corticotomy technique (tibia), a 5–10 mm longitudinal incision over the lateral border of the tibia is made, and the periosteum is elevated. A 5-mm osteotome is used to cut and twisted to spread the periosteum. The anterior half of the lateral tibial cortex is then osteotomised, after which the medial periosteum is elevated. Following this, the medial cortex of the tibia is osteotomised, under the protection of the elevator. Likewise, the remaining lateral cortex is divided, once again under the protection of the elevator. The osteotomy is seen to completion by a rotational osteoclasis typically requiring the Ilizarov frame to have been applied beforehand, with no connecting rods linking the rings adjacent to the osteotomy (Fig. 2) [3].

Rotation completes the osteotomy across the posterior tibial cortex, but in a rather unpredictable fashion. There is a risk the osteotomy line can propagate towards wire and pin sites and thus these must be sufficiently far enough away to reduce this risk.

## De Bastiani technique: ‘multiple drill hole osteotomy’

During the 1980’s, multiple drill osteotomy technique was then introduced which only required a small incision and was found to be more precise than the corticotomy technique. It was popularised by the Verona group, namely De Bastiani et al., later recognised as the De Bastiani technique, which can be applied to any long bone. The corticotomy was performed in the proximal area of the diaphysis utilising an anterior approach in all cases. Careful blunt dissection of the periosteum was performed and achieved using a 4.8 mm diameter drill piece in a short screw guide. To prevent damage to the medullary cavity and bone marrow, a stop on the drill was adjusted so that no more than 1.0 cm of drill was projecting beyond the end of the guide [40].

**Fig. 3:**
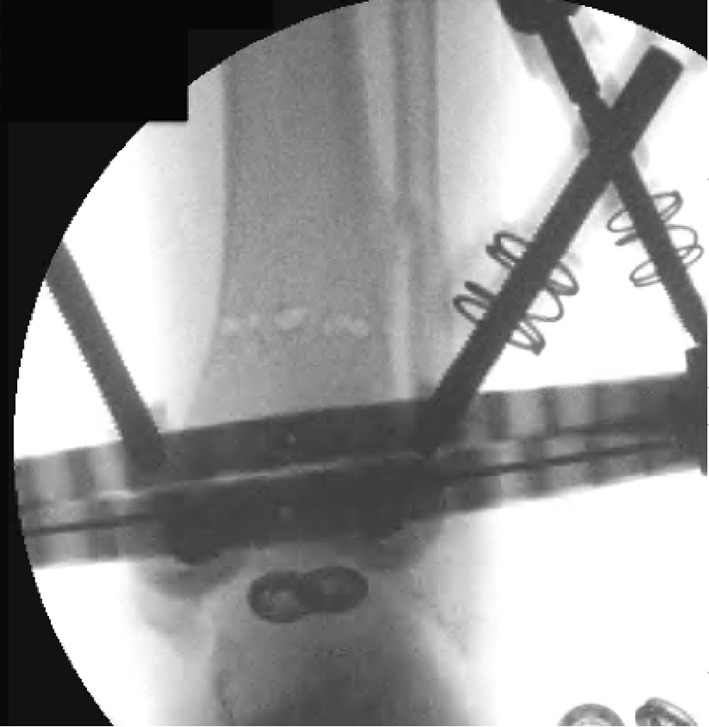
Clinical radiograph demonstrating the appearance of the distal tibia following the result of the multiple drill hole osteotomy being performed

**Fig. 4:**
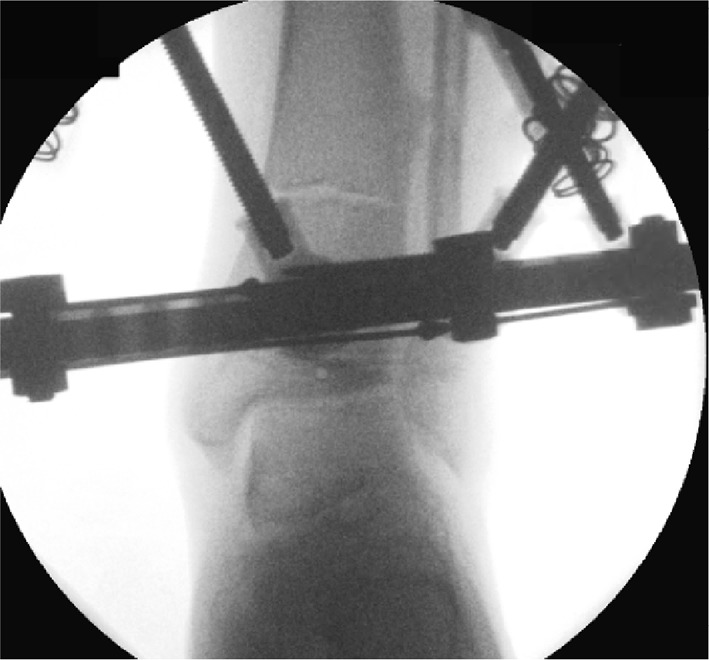
Appearance of the tibia following the connection of the multiple drill holes with an osteotome. This will complete the osteotomy in the tibia

**Fig. 5:**
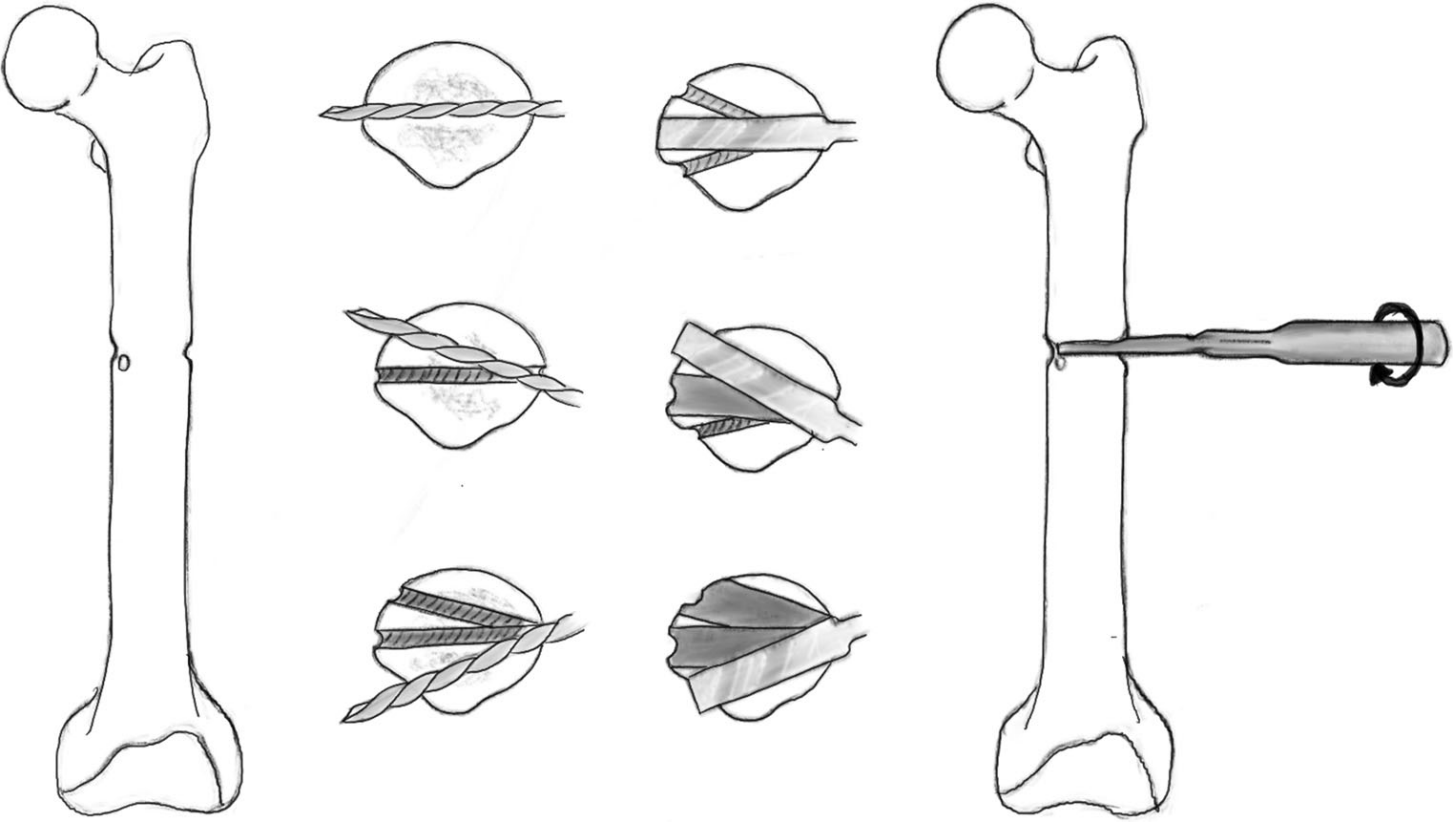
Diagrammatic (coronal and axial) sequential representation of the direction of the multiple drill hole osteotomy technique. Redrawn from original illustration in Principles of Deformity Correction, Paley [3], Springer

**Fig. 6:**
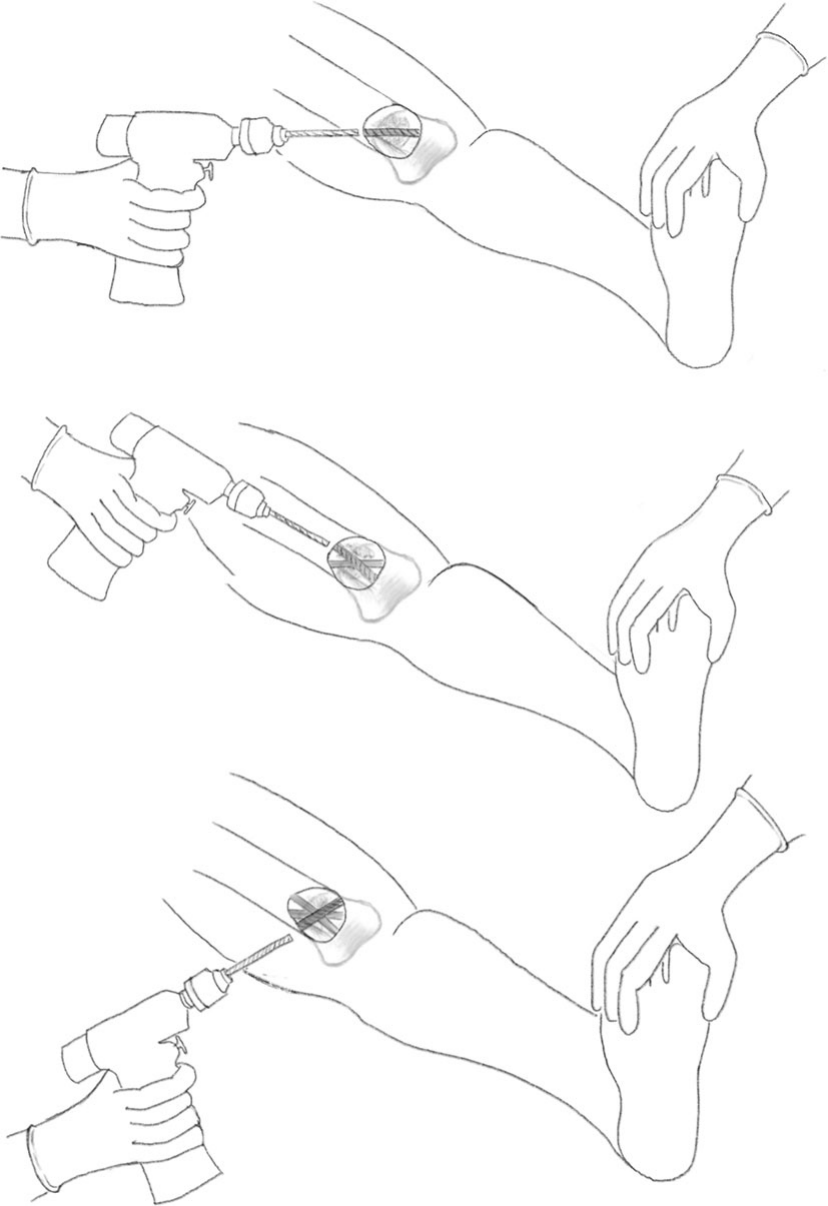
When using a drill to facilitate the multiple drill hole technique, the drill trajectory can be altered to osteotomise the bone

**Fig. 7:**
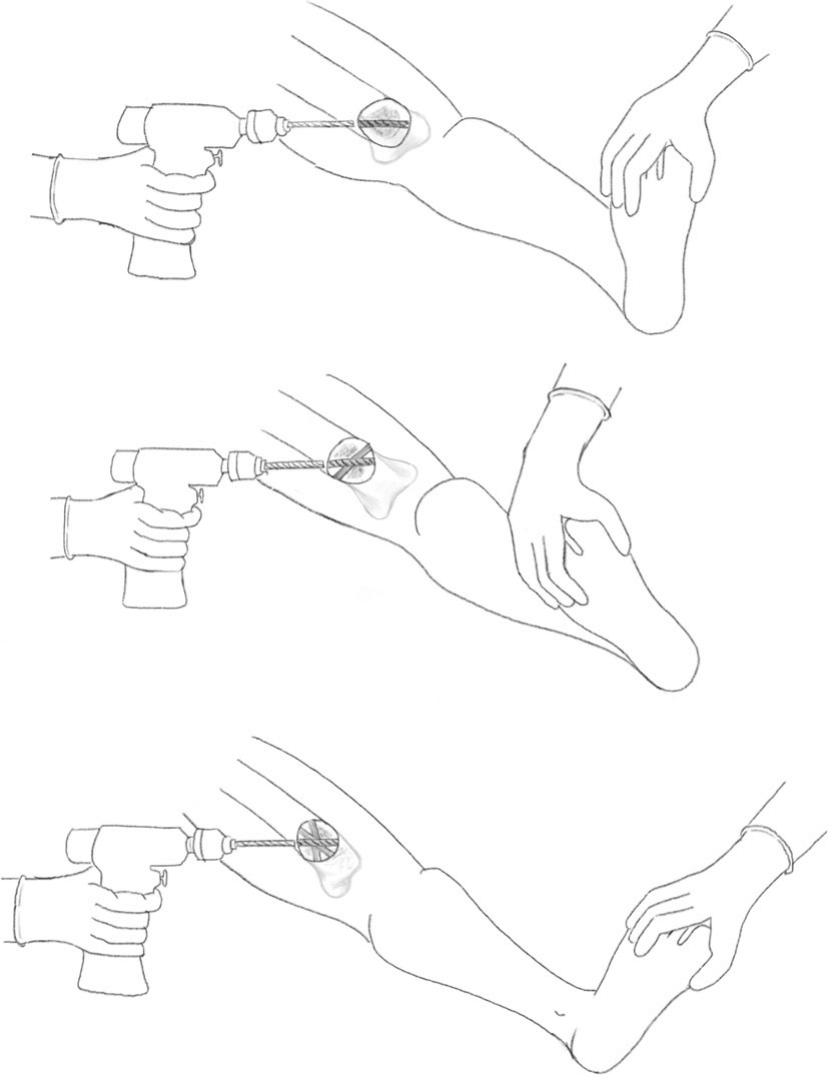
Illustration showing rotational osteoclasis of posterior cortex following corticotomy of anteromedial and anterolateral cortex

A series of holes are then drilled around the anterior two-thirds of the bone circumference; the first drill hole was lateral to medial, the second was redirected in an oblique anteromedial direction, and subsequently to a posteromedial direction (Figs. 3, 4). Following the multiple drill holes, an osteotome then connects the drill holes to complete the osteotomy (Fig. 5). The posterior cortex was automatically broken, which meant the rear periosteum remained intact; this protects the integrity of the posterior periosteum. The medullary vascularity can be damaged during this process despite measures to protect it; however, it recovers within a few days [41]. There are different ways to achieve the desired orientation of the drill holes. The bone to be osteotomised should be stabilised and the drill trajectory adjusted by the operator (Fig. 6), or the trajectory of the drill should be stabilised by the operator and the position of the limb rotated to achieve the desired drill holes (Fig. 7).

This technique was then advanced and performed percutaneously. Performing this osteotomy method is less technically demanding and has an easier learning curve than other more complex osteotomy techniques, such as the Gigli saw osteotomy. The periosteum can be preserved whilst performing the multiple drill osteotomy, which has been shown to be integral in gap regeneration during distraction osteogenesis.

## Afghan technique: ‘Gigli saw osteotomy’

**Fig. 8:**
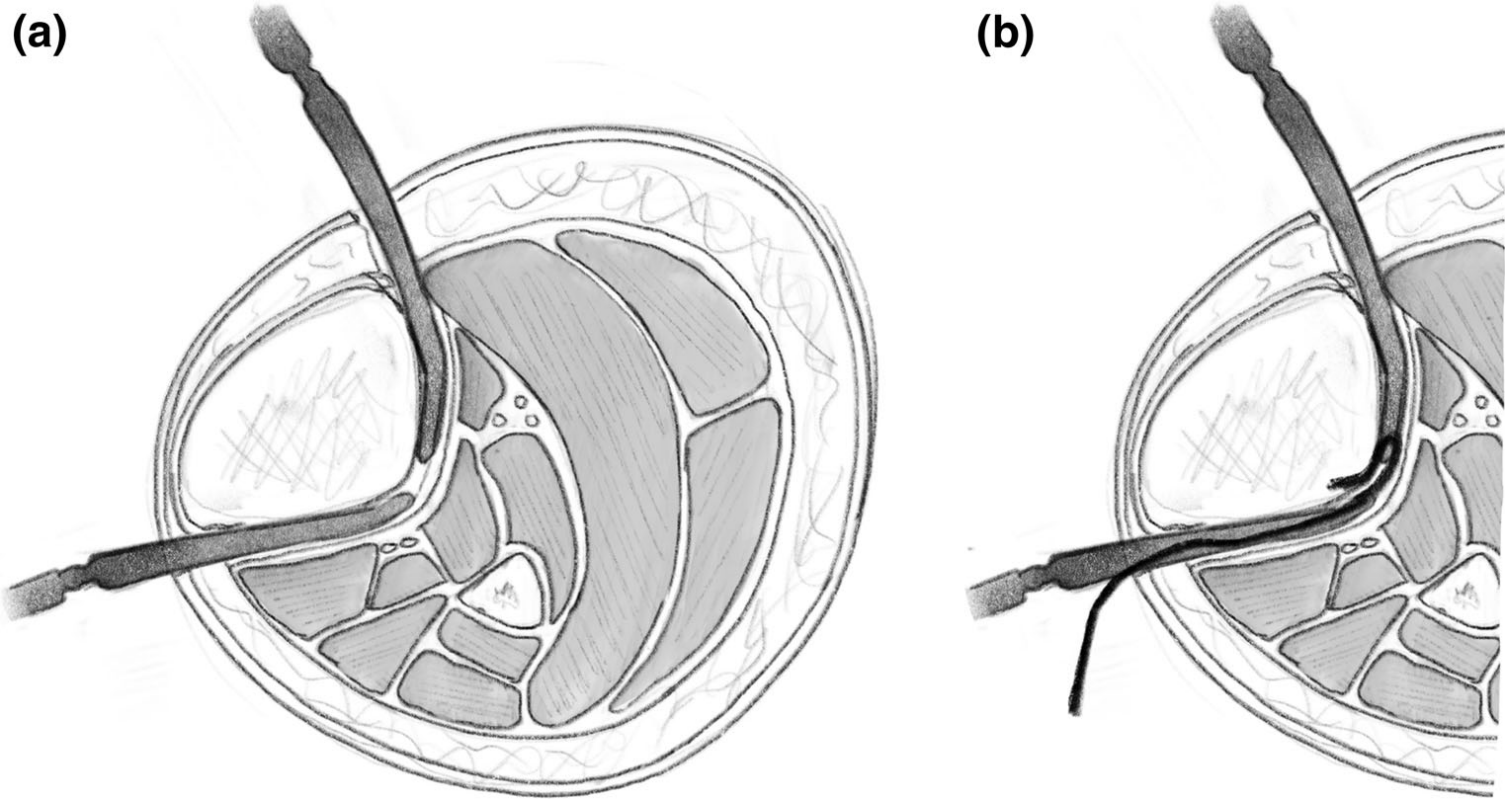
**a** A blunt elevator is used to create a subperiosteal tunnel around the bone to be osteotomised. The periosteum is elevated to avoid penetration of the fascial compartment and protect the neurovascular structures. **b** A suture is then passed in this subperiosteal tunnel and the Gigli saw is introduced and passed carefully. Redrawn from original illustration in Principles of Deformity Correction, Paley [3], Springer

The percutaneous Gigli saw technique, also known as the Afghan technique, is a recognised and popular method for performing osteotomies in long bones, as well as the foot. It should be avoided when there is thick diaphyseal cortical bone and preferentially used in metaphyseal area. Here, two transverse incisions are made; subperiosteal tunnels are created with a right angle and curved clamp from a posteromedial to anterolateral direction (Fig. 8a). The Gigli saw is then tied to the suture and pulled through from posterior to anterior (Fig. 8b). The posteromedial aspect of the tibia creates a sharp bend; hence, it may be beneficial to create a small bend in the Gigli saw to allow easy passage [3].

**Fig. 9:**
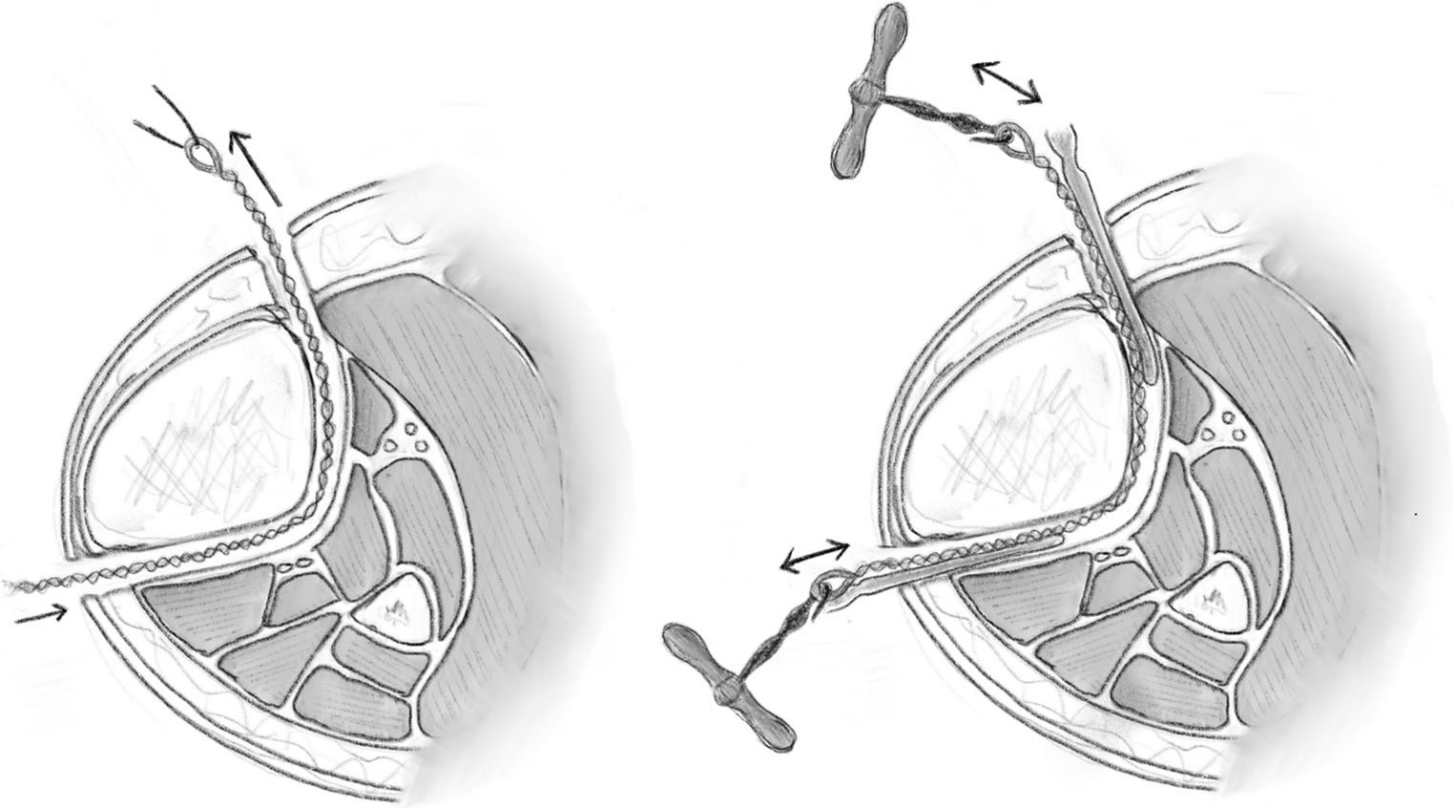
After the Gigli saw is positioned, the lateral and posterior cortices can be cut under the protection of the elevator. Redrawn from original illustration in Principles of Deformity Correction, Paley [3], Springer

**Fig. 10:**
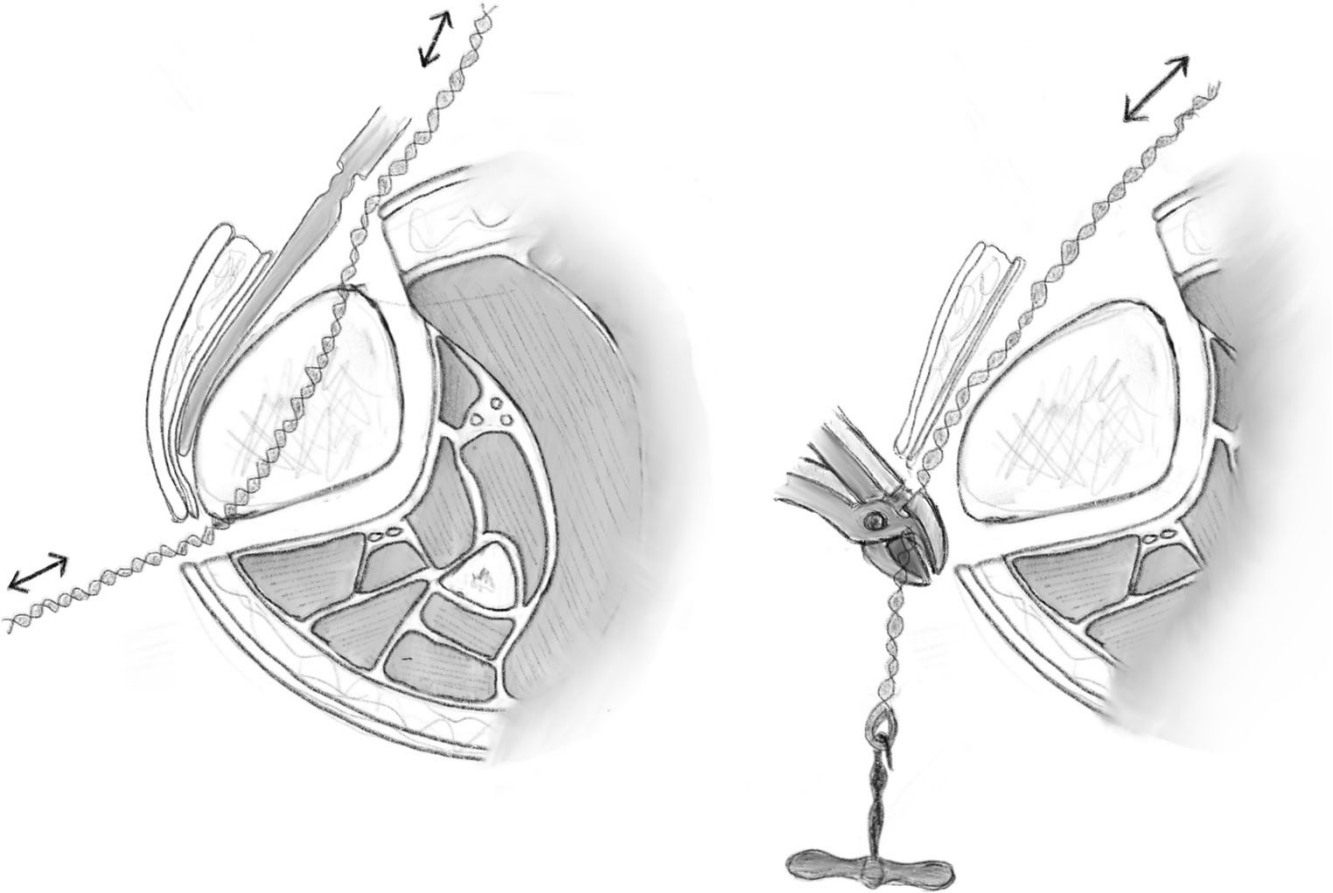
The medial cortex is cut, once again under the protection of the elevator as it passes under the medial periosteum. Redrawn from original illustration in Principles of Deformity Correction, Paley [3], Springer

The posterior and lateral cortices are cut with the Gigli saw under the protection of the elevators (Fig. 9). The medial cortex periosteum is then elevated, and the flattening out the direction of the cut with the Gigli saw cuts the medial cortex (Fig. 10). There is minimal periosteal disruption and limited concern of thermal necrosis. In addition, the soft tissue envelope is not breached which aids the periosteal blood supply.

Gigli saw osteotomy is a low-energy osteotomy that leaves a very smooth cut, which is especially important for rotational correction [42]. Another advantage of this method is that the surgeon can first perform an incomplete osteotomy, and then complete it after the application of the external fixation device. This is of benefit as it is easier to apply a fixator to a stable bone. The last few millimetres are cut at the end of the operation. Moreover, this can be done completely percutaneously and subperiosteally producing a precise osteotomy.

There are numerous other advantages for the Gigli saw technique, including that there is no need to disassemble the frame and hence can be performed with minimal interruption of the circular frame. This method creates a very neat fracture line, confirming the definitive completion of the osteotomy, once through the cortex. The risks associated with the Gigli saw could be an injury as a result of cutting soft tissues around the bone. Consequently, this led to the design and production of similar alternative methods being created such as the Threadwire saw [43].

## Dome osteotomy

The dome osteotomy is a circular-shaped bone cut. The CORA (centre of rotation of angulation) corresponds to the centre of the circular cut and the point of rotation for the dome. There are limitations to be considered prior to performing the Dome osteotomy, including the radii of the circular cut. The larger the radii, the more translation will occur, hence the less bone-to-bone contact and ultimately less stability for the fixation method of choice. The Dome osteotomy itself does not pass through the CORA. The bone ends at the osteotomy line must angulate and translate, producing a secondary translational deformity. Performing these osteotomies in metaphyseal bone is most practical as this provides the widest diameter of bone.

**Fig. 11:**
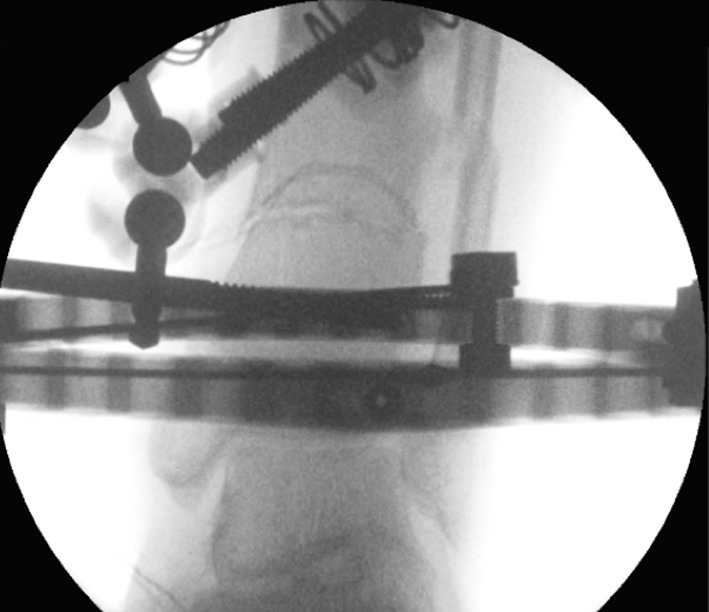
Clinical radiographs representing examples of the dome osteotomy in a distal tibia with a circular frame in situ

**Fig. 12:**
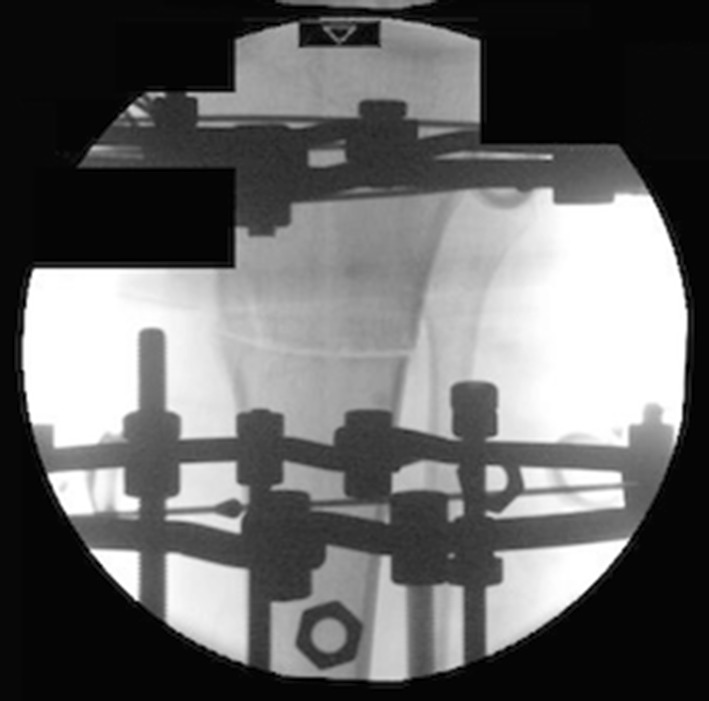
Clinical radiographs representing examples of the dome osteotomy in a proximal tibia with a circular frame in situ

After the Dome osteotomy has been performed, one of the advantages is large bone-to-bone contact and stability. It is still a version of a drill and osteotome osteotomy, but is technically more challenging (Figs. 11, 12). Disadvantages of the procedure include difficulty with rotational corrections.

Dome osteotomies can be used to correct angular deformities but not axial rotation, although modifications of the dome can be made to correct angulation as well as rotation. One way would be to incline the dome cut, inclining the axis of correction. Alternatively, a spiral dome can be used. There are several ways to complete this osteotomy. Special curved saws and osteotomes are available for small bones; larger bones may require multiple drill holes in a circular pattern and completed with an osteotome. It is important to use the CORA as the point of rotation. The more complex dome’s are technically challenging and may not realistically be practical in all soft tissue envelopes.

## Power saw osteotomy

Power saw osteotomies require a relatively large open exposure, and thus have disadvantages of soft tissue stripping around the osteotomy site. These instruments can cause thermal necrosis of the bone, but this can be prevented by irrigation of the saw blade with cold saline. This technique is used in closing wedge osteotomies, forming perfectly coated surfaces, which allow for compression and stability. There is very good healing potential with a large bone-to-bone contact area and an almost absent wedge volume.

**Fig. 13:**
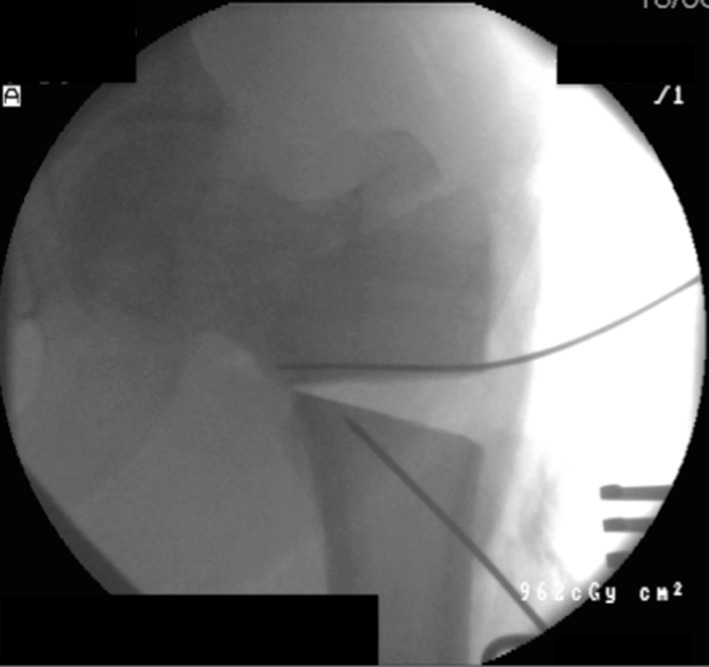
This radiograph demonstrates a proximal femoral Pauwels osteotomy, which was first introduced in 1927. It is an osteotomy, which reorientates shear forces to compressive forces. This is essentially a closing wedge osteotomy

**Fig. 14:**
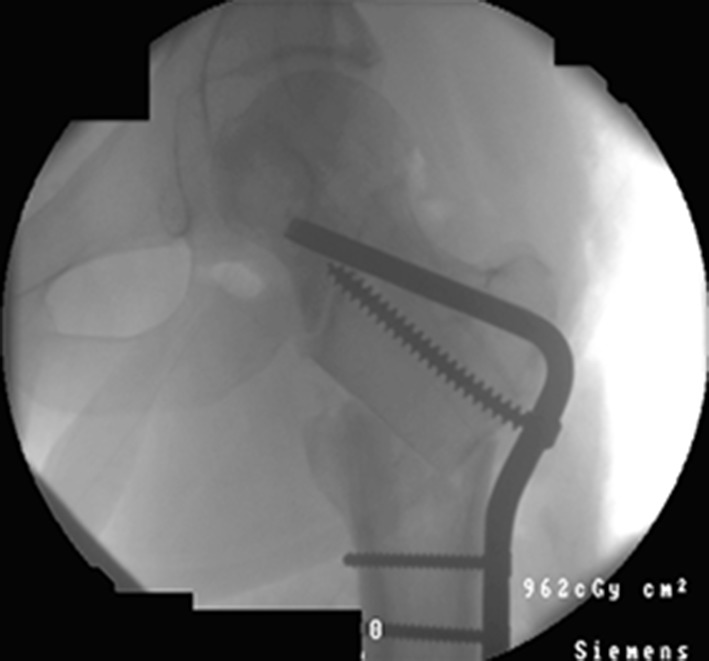
Closing wedge osteotomies are performed using a saw blade. In this example, the femoral neck is realigned into valgus to achieve compressive properties at the fracture site

Closing wedge osteotomy is a very popular technique performed in orthopaedic surgery due to the excellent bone-to-bone contact and stability (Figs. 13, 14). This technique is usually performed as an open procedure, under direct vision, as a wedge is excised. The closing wedge is stabilised with internal fixation usually with screws and plates. Such examples are when plates for proximal tibial osteotomies are used to treat osteoarthritis of the knee and blade plates for the proximal femur. When the CORA is at the apex of the wedge, no secondary translation will occur. Each osteotomy of the closing wedge should be made perpendicular to the long axis of each respective side of bone segment. A common difficulty occurs when the closing wedge cuts are not parallel with the long axis of the bone, ultimately leading to the generation of a shear force when the opposing sides are compressed. Additionally, if the plane of the cuts is different, bone-to-bone contact becomes a serious issue. Pre-operative templating and thickness of blades and saws can further add to the inaccuracies of this method and hence complications.

One major concern with opening wedge osteotomies is the risk of bone healing due to lack of bone-to-bone contact. Hence, graft has been used for structural support with success.

## Relevance of blood supply in osteotomy

The ideal situation following a corticotomy will be the environment of an undisplaced fracture. This ideal setting preserves the medullary and periosteal blood supply [44]. Both sources of blood supply have a role in distraction osteogenesis, the importance of which is still under debate. The goal of the corticotomy is to preserve the endosteal and periosteal supply, which is very technically challenging.

All osteotomies performed through an extensile approach will cause some devascularisation of bone; the dissection of the periosteum should be kept to minimum. Circulation to the bone must be considered as the high-pressure nutrient arterial system supplies the inner 2/3 of the bone, whereas the outer 1/3 is supplied by the low-pressure periosteal system. When an osteotomy is performed, the blood supply will change from a centrifugal to a centripetal flow, rendering the low-pressure periosteal system predominant.

## Distraction osteogenesis and bone regeneration

Distraction has previously been regarded as a factor contributing to non-union by interposition of fibrous tissue [45]. As introduced by Ilizarov, gradual mechanical distraction of a low-energy osteotomy spontaneously produces potentially unlimited new bone from the local host bone. This rapidly remodels to normal structure, even in skeletally mature bone [46]. Ilizarov claimed the process of osseous regeneration is enhanced provided the environment was stable, the preservation of bone vascular supply and weight bearing were all combined [47].

Ilizarov introduced distraction osteogenesis by chance [46]. A case of hypertrophic non-union was supposed to be treated with compression, however the nuts on the rods were turned in the wrong direction creating distraction forces instead of the intended compression.

Numerous studies have subsequently been performed evaluating the regenerative properties of different osteotomies to establish which osteotomy technique yields the best osseous regenerate and the importance of the blood supply on the new bone formation. Delloye et al. performed an experimental study, using adult female mongrel dogs, assessing the pattern of bone regeneration from cortical bone segments during distraction lengthening. They found no difference in pattern of healing and the amount of newly formed bone after corticotomy or osteotomy, i.e. no difference in preserving medullary or periosteal blood supply. They did find, however, that the stability, rate of continuous distraction and function of the limb were more important factors in osseous regeneration [48]. Delloye’s experimental work did not demonstrate a difference in bone regenerate when different osteotomies were performed. Others later challenged this theory.

Kojimoto et al. expressed after his experimental studies on 27 growing rabbits that the periosteal system preservation was more important than the endosteal, and that the preservation of the periosteum was in fact more important than careful osteotomy [49]. De Bastiani and Ilizarov had recommended careful limited corticotomy to protect the bone marrow and ultimately medullary blood supply. Kojimoto demonstrated the endosteum and bone marrow were not indispensible for adequate callus formation and the periosteum was in fact an important contributor to osseous regeneration.

Brutscher et al. conducted an experimental study on sheep tibiae to assess the differences in regenerative properties when the division was by either a corticotomy or osteotomy. At 8 weeks after the procedure, there was no remarkable difference between the cases of corticotomy and osteotomy. From the 9th week onwards, bone regeneration after osteotomy was considerably delayed and even more so after the 12th week [50]. Brutscher highlighted several factors to consider in their work. The feasibility of the corticotomy relied on the condition of the medulla and cortex, instruments available and the experience of the surgeon. The work concluded that the corticotomy group developed new tubular bone quicker and with fewer complications and was seen as optimal. The osteotomy group led to regenerate, which was not tubular initially, however, did lead to tubular bone after remodelling. Once again the importance of the medullary circulation was highlighted.

Frierson et al. conducted a study comparing three different osteotomy techniques in assessment of regeneration properties in distraction osteogenesis. Their findings showed that vessels bridging the regenerate gap were diminished in the group in which the oscillating saw was used to perform the osteotomy. However, there was an abundance of vessels bridging the gap in the remaining two groups, corticotomy and multiple drill hole groups. This then led them to conclude that the oscillating saw may lead to delayed consolidation [51]. The challenge of keeping the medullary endosteal supply in continuity during the corticotomy has surfaced repeatedly [22, 29]. Dividing the anteromedial and anterolateral cortices through an anterior approach is relatively simple. To complete the corticotomy, a rotational force is applied. This frequently results in an oblique fracture through the posterior cortex as it is the central axis of rotation. The medullary contents are said to undergo shearing that disrupt the vascular supply. Frierson, Kojimoto, Kawamura and Aronson all confirmed neovascularisation of arterioles following transection of the medullary blood supply. Frierson’s work also confirmed osseous regeneration in distraction can be achieved following complete transection of the medullary vascular supply. In addition, there were no histological or radiological differences between the corticotomy and transverse osteotomy groups.

These animal studies have established several important aspects in distraction osteogenesis. Ilizarov’s concept of maintaining a stable fixation and environment with full weight bearing is critical as well as the vascular supply. The importance of the endosteal and medullary supply, however, has undergone debate, and these studies have shown that the multiple drill hole osteotomy helps prevent comminution and fracture line propagation. It is safe and technically straightforward. Rapid neovascularisation of the medullary vascularity does occur and care needs to be taken to protect the periosteum when performing this procedure.

## Overview of methods

Osteotomies differ in the extent of surgical exposure necessary to complete them. With larger exposure and greater dissection, soft tissue stripping can occur and damage to the vital periosteum and ultimately vascular supply to the osteotomy can occur. Percutaneous techniques have been developed but do carry a higher risk of injury to vascular and neurological tissue. The deleterious effects of open exposure can be diminished nevertheless, by preserving the periosteum and soft tissue sleeve surrounding the bone.

We know from Ilizarov’s experience the problems encountered with the classical open osteotomy using Power saws, such as the oscillating saw, which causes thermal necrosis of bone tissue. Frierson also confirmed that when the power saw is used to perform the osteotomy for distraction osteogenesis, there is decreased bridging vessels and radiographically wider lucent centres throughout the distraction period. The Gigli saw technique and the Drill and Osteotome osteotomy both preserve the periosteal blood supply. They are minimally invasive and are low-energy procedures [52].

The original corticotomy is not recommended for perfect and well-localised bone interruption in case of bone transport, where bone segments are short and fragile. Hence the multiple drill hole technique is the best technique for distraction osteogenesis within this context [40].

The energy to divide the bone is an additional factor that influences the viability of the osteotomy site and its osteogenic potential. Power saws and high-speed burrs can cause thermal necrosis of the bone ends and adjacent soft tissues, though these are still used.

New bone formation is more rapid with the original corticotomy and multiple drill hole osteotomy than the Gigli saw, so long that the latency period between the day of the osteotomy and the day of lengthening is the same. In the Gigli saw osteotomy, if the latency period is longer than 15 days the new bone formation is more evident and homogenous [40].

An experimental model in sheep looked at five different osteotomy techniques ranging from subcutaneous corticotomy to open osteotomy and their effect on new bone formation. It found that osteoclasia using multiple subcutaneous drilling to allow subsequent fracture gave the most advanced remodelling and mineralisation of bone tissue [53]. Each of these techniques pose varying degrees of damage to the periosteum and bone marrow. Interestingly, the techniques both preserving and damaging the bone marrow resulted in comparable regenerates; total damage to the periosteum and preservation of the medullary supply inhibited bone regeneration during distraction. The classical subcutaneous osteotomy described by Ilizarov has been shown by numerous studies [51, 53] to be most traumatic and poses a real challenge to preserving the periosteum and soft tissues around the bone. The preservation of the periosteal sleeve in the multiple drill hole technique provides strong induction properties of osteogenic cells. Shavings, within the gap as a result of the multiple drill holes, contain polypeptide growth factors (Bone Morphogenic Proteins and Transforming Growth Factor Beta) which are signals inducing proliferation of undifferentiated mesenchymal cells of periosteum and endosteum towards osteogenic cells. This method, in spite of damage to the bone marrow, yielded a quicker formation of bone regenerate.

Without clinical trials, it is not possible to accurately conclude which osteotomy method yields the best bone regenerate. Animal and basic science studies have investigated this, as discussed; however, one must hold reservations extrapolating this evidence and its application in human biology. There are conflicting reports in the literature, and it is not possible to draw firm conclusions from the evidence base.

One of the advantages of extrapolating data from animal studies is that there is an abundance of animal experimental data in the literature. Although the genetic make-up of humans and animals are not identical, there are similarities within the skeletal constitution. Animal studies can be indicative of positive findings but certainly cannot be conclusive. One of the limitations of interpreting animal models is that the mechanical environment regardless of the stabilisation technique will not be identical to the human–environment, such as the body mass and load through the osteotomy. These variables make interpretations of animal studies inconclusive.

## Conclusion

Our approach to performing an osteotomy is based on many factors including previous research, current literature and our own surgical experience, where regenerative properties for each osteotomy and variants of correction are continually considered. On the one hand, ‘Drill and Osteotome’ osteotomy is the most commonly practiced technique, offering adequate regenerative properties and is the most favourable in our experience for performing distraction osteogenesis. In opposition, the Gigli saw yields a clean and organised osteotomy, though the regenerative properties are not as profound. The ‘drill and osteotome’ technique, when performed percutaneously, is our preferred method as there is no formal exposure of the periosteum. With low speed, cooled drilling and limited drill passes, the risk of adverse thermal necrosis with this method is kept to a minimum. Being able to perform this osteotomy whilst not making any gross changes to the frame construct or the post-operative rehabilitation is also another major advantage of this technique.
